# Artificial Intelligence-Based Evaluation of Permanent First Molar Extraction Indications in Children Using Panoramic Radiographs

**DOI:** 10.3390/children13020277

**Published:** 2026-02-17

**Authors:** Serap Gülçin Çetin, Ömer Faruk Ertuğrul, Nursezen Kavasoğlu, Veysel Eratilla

**Affiliations:** 1 Faculty of Dentistry, Batman University, 72100 Batman, Turkey; serapgulcincetin@gmail.com (S.G.Ç.); veyseleratilla@gmail.com (V.E.); 2 Faculty of Electrical and Electronic Engineering, Batman University, 72100 Batman, Turkey; omerfaruk.ertugrul@batman.edu

**Keywords:** permanent first molar, dental age assessment, Demirjian method, panoramic radiography, artificial intelligence, machine learning, clinical decision support

## Abstract

**Highlights:**

**What are the main findings?**

- An artificial intelligence-based Gabor–HOG–SVM model can classify permanent first molar extraction indications in children using panoramic radiographs with acceptable accuracy.
- The model demonstrated balanced performance between extraction-indicated and non-indicated cases without age- or sex-related bias.

**What are the implications of the main findings?**

- AI-assisted analysis of panoramic radiographs may support clinicians by reducing observer-dependent variability in pediatric extraction decisions.
- The proposed approach provides a reproducible decision-support framework that can be expanded and validated in multicenter pediatric dental studies.

**Abstract:**

**Background:** The aim of this study was to develop an artificial intelligence (AI)-based decision support model for evaluating the extraction indication of permanent first molars in pediatric patients using panoramic radiographs, and to investigate the potential contribution of this model to the clinical decision-making process. **Methods:** This retrospective observational study analyzed 1000 panoramic radiographs obtained from children aged 8–10 years who attended the Clinics of Batman University Faculty of Dentistry for routine dental examination. Among the radiographs meeting the inclusion criteria, a total of 176 panoramic images were selected based on dental maturation assessment using the Demirjian tooth development staging system. Cases in which the permanent second molar was classified as Demirjian stages E–F were labeled as “extraction indication present”, while the remaining stages were labeled as “extraction indication absent”. A balanced dataset was created, consisting of 88 cases in each group. Image features were extracted using Gabor filters and Histogram of Oriented Gradients (HOG). The selected features were analyzed using a Support Vector Machine (SVM) classifier with a radial basis function (RBF) kernel. Model performance was evaluated using accuracy, sensitivity, specificity, F1-score, and area under the receiver operating characteristic curve (ROC–AUC). **Results:** The proposed Gabor–HOG–SVM-based AI model achieved an overall classification accuracy of 77.78% with an AUC value of 0.77 in distinguishing between “extraction indication present” and “extraction indication absent” cases. For the extraction-indicated group, the sensitivity was 0.81 and the F1-score was 0.79, whereas for the non-indicated group, the sensitivity and F1-score were 0.74 and 0.77, respectively. No statistically significant differences were observed between the groups in terms of age or sex distribution (*p* > 0.05). **Conclusions:** This study demonstrates that artificial intelligence-based analysis of panoramic radiographic images can provide an objective and reproducible decision support approach for evaluating extraction indications of permanent first molars in pediatric patients. The proposed model should be considered as an adjunctive tool to reduce observer-dependent variability rather than a replacement for clinical judgment, and its clinical applicability should be further validated through multicenter and multi-parametric studies.

## 1. Introduction

Clinical decision-making in pediatric dentistry frequently requires the integration of multiple clinical, developmental, and radiographic parameters, particularly when treatment decisions may influence future occlusion and craniofacial growth. Panoramic radiography remains one of the most commonly used imaging modalities for evaluating dental development and eruption patterns in children; however, its interpretation is inherently subject to observer-related variability, positioning errors, and differences in clinical experience, which may affect diagnostic consistency and treatment planning outcomes [1].

In recent years, artificial intelligence (AI) has emerged as a powerful tool across various medical and dental fields, including diagnostic imaging, offering the potential to improve objectivity, standardization, and reproducibility in image-based assessments [2]. Importantly, contemporary AI applications in healthcare are not intended to replace clinical expertise, but rather to function as supportive decision-making tools that augment clinician judgment and reduce subjective variability [3]. Within this framework, AI-assisted analysis of panoramic radiographs may provide valuable support in complex pediatric dental decisions, such as determining extraction indications for permanent first molars.

Permanent first molars are the first permanent teeth to erupt into the oral cavity and represent the onset of the mixed dentition period. Due to their early eruption timing and complex morphological characteristics, they are among the teeth most susceptible to dental caries in the permanent dentition. This vulnerability may negatively affect their prognosis and lead to premature tooth loss during childhood. Nevertheless, permanent first molars play a critical role in maintaining occlusal continuity, masticatory efficiency, and vertical facial height. Therefore, preservation of these teeth and their accurate clinical evaluation are of great importance in pediatric dental care [4].

Etiological factors such as dietary habits, inadequate oral hygiene, systemic and environmental influences, and developmental enamel and dentin defects may adversely affect the prognosis of permanent first molars. In this context, determining the indications and optimal timing for extraction of these teeth—often referred to as the key to occlusion—is of critical clinical importance. Untimely or uncontrolled extractions may result in tipping, migration, or overeruption of adjacent teeth, leading to occlusal disturbances. In contrast, it has been reported that when extractions are performed at an appropriate time, the second and subsequently the third molars may move mesially into the extraction space more favorably, thereby facilitating the establishment of a functional occlusal relationship [5].

The literature indicates that the ideal timing for extraction of permanent first molars is generally between 8 and 10 years of age, corresponding radiographically to the stage at which crown formation of the second molar is complete and approximately one-third of root development has occurred [6]. This developmental stage is consistent with Demirjian stage E in the dental maturation system [7]. Therefore, accurate prediction of the prognosis of permanent first molars and optimal planning of extraction timing require a comprehensive assessment combining clinical findings with radiographic evaluation.

Radiological imaging plays a fundamental role in diagnosis, treatment planning, and follow-up in dentistry. Panoramic radiographs are widely used imaging modalities that allow monitoring of the calcification processes of permanent first and second molars, as well as providing an overall assessment of the maxillomandibular complex and dentoalveolar structures [8]. However, observer-dependent variability in the interpretation of radiographic findings may limit the objectivity and reproducibility of diagnostic assessments.

In recent years, AI technologies have gained increasing prominence in dentistry, as in many other medical disciplines. AI-based systems have been successfully applied in dental radiology for various clinical purposes, including detection of carious lesions, evaluation of periodontal disease, identification of the mandibular canal trajectory, prediction of skeletal growth, and detection of cystic or neoplastic lesions [9,10,11,12]. These advancements enable more objective, standardized, and reproducible analysis of radiographic data.

Despite these developments, determining the extraction indication for permanent first molars remains a significant clinical challenge. The need to simultaneously consider factors such as extent of carious involvement, restorability of the tooth, patient age, dental and skeletal development, and radiographic findings complicates the decision-making process. Subjective evaluations based on clinical experience may lead to variability in extraction decisions, potentially increasing the risk of long-term occlusal disturbances and subsequent orthodontic treatment needs.

In this context, AI-based clinical decision support systems, capable of evaluating multiple clinical and radiographic parameters simultaneously, have the potential to offer substantial advantages to clinicians in determining extraction indications. However, the current literature lacks a standardized and validated AI-based system that evaluates extraction decisions for permanent first molars in pediatric patients using panoramic radiographs.

AI-based systems have demonstrated increasing utility across a wide range of medical and dental applications, particularly in diagnostic imaging and pattern recognition tasks. By enabling the automated analysis of complex image data, AI has the potential to enhance diagnostic consistency, reduce observer-dependent variability, and support clinicians in managing multifactorial decision-making processes. However, it is essential to emphasize that AI-driven tools are designed to complement, rather than replace, clinical expertise. In pediatric dentistry, where treatment decisions may have long-term implications for growth and occlusal development, AI-assisted decision support systems should be regarded as adjunctive tools that aid clinicians in making more objective and reproducible assessments [2,3].

Although AI has been increasingly applied to panoramic radiographs for tasks such as tooth detection, segmentation, and dental development or age assessment, clinically oriented AI models focusing specifically on extraction decision support remain scarce. Most existing studies aim to identify anatomical structures or classify developmental stages rather than to assist clinicians in complex treatment-related decisions. In this context, the present study is positioned to address a distinct clinical gap by proposing an AI-based decision-support framework specifically designed to evaluate the optimal timing window for permanent first molar extraction in pediatric patients using panoramic radiographs.

Therefore, the aim of this study was to develop an AI-based evaluation model using data derived from panoramic radiographs to determine timing-based extraction indications for permanent first molars in children and to investigate the contribution of this model to the clinical decision-making process.

The null hypothesis of this study was that there would be no statistically significant difference between extraction decisions obtained using the AI-assisted evaluation model and those derived from conventional clinical assessment methods.

## 2. Materials and Methods

### 2.1. Study Design and Ethical Approval

This study was designed as a retrospective, observational investigation aimed at evaluating the extraction indication of permanent first molars in pediatric patients using panoramic radiographs. The panoramic radiographs analyzed in this study were obtained from children who attended the Clinics of Batman University Faculty of Dentistry for routine dental examination and treatment purposes.

The study protocol was approved by the Ethics Committee of the Faculty of Dentistry, Batman University (Approval No: 2026/01-3). All procedures were conducted in accordance with the principles of the Declaration of Helsinki, and written informed consent was obtained from the legal guardians/parents of all patients prior to the use of radiographic data. All data were anonymized before analysis.

### 2.2. Study Group and Demographic Characteristics

Within the scope of this retrospective study, a total of 1000 panoramic radiographs were initially screened. Among these, 176 panoramic images meeting the predefined inclusion criteria were selected for AI model development. To ensure class balance, the final dataset consisted of 88 cases with extraction indication and 88 cases without extraction indication.

The study group comprised 82 boys and 94 girls, all in the mixed dentition period. To enhance comparability between classes and prevent model bias, the dataset was deliberately structured in a balanced manner with equal representation of extraction-indicated and non-indicated cases.

The age range of 8–10 years was selected based on established developmental and clinical guidelines for the extraction timing of permanent first molars. This period generally corresponds to the Demirjian stage E–F of the permanent second molar, during which crown formation is complete and initial root development has begun. Extraction performed within this developmental window has been associated with more favorable spontaneous mesial migration of the second molar and improved occlusal outcomes. Therefore, chronological age was used as a practical surrogate for underlying dental maturation rather than as an isolated selection criterion [13,14].

Given the exploratory and pilot nature of this AI- based study, sample size determination was not based on conventional hypothesis-driven power calculations. Instead, a balanced dataset was constructed to optimize classifier training and performance evaluation. The selected sample size is consistent with previously published pilot AI studies utilizing panoramic radiographs and classical machine learning methodologies.

### 2.3. Imaging Protocol

All panoramic radiographs were acquired in the same clinical setting, using a standardized imaging protocol and the same panoramic radiography device (PCH-2500, VATECH Co., Ltd., Hwaseong-si, Republic of Korea). A single-center, single-device imaging approach was adopted to minimize potential variations arising from inter-device technical differences.

Radiographs exhibiting significant motion artifacts, distortion, or insufficient image quality were excluded from the study.

### 2.4. Reference Assessment and Demirjian-Based Labeling

To establish an objective and standardized reference for determining the timing of extraction indication for permanent first molars, the developmental stage of the permanent second molar in the relevant quadrant was assessed on panoramic radiographs using the Demirjian tooth development staging system.

According to the literature, the most favorable time window for extraction of permanent first molars in the mandible corresponds to the period between Demirjian stage E, when bifurcation mineralization of the permanent second molar begins, and Demirjian stage F, when root development progresses further. Accordingly, cases classified as Demirjian stages E–F were considered to fall within the appropriate extraction window and were labeled as “Indication present”. Cases outside these stages were classified as “Normal (no extraction indication)”.

All panoramic radiographs were independently evaluated by two experienced examiners for dental maturation assessment using the Demirjian tooth development staging system. Both examiners were calibrated prior to the evaluation process and were blinded to each other’s assessments as well as to the final classification labels. In cases of disagreement, a consensus was reached through joint re-evaluation.

Interobserver agreement was assessed using Cohen’s kappa (κ) coefficient and demonstrated a high level of agreement between the two evaluators (κ = 0.82), indicating that the Demirjian-based reference labeling was reliable and reproducible.

As a result, a balanced dataset consisting of Indication (*n* = 88) and Normal (*n* = 88) cases was constructed, and these labels were used as the ground truth during training and evaluation of the AI model. Importantly, the reference labeling in this study represents a timing-based extraction indication window derived from dental developmental stages rather than a comprehensive clinical extraction decision.

### 2.5. Image Data and Preprocessing

All panoramic radiographs underwent a standardized preprocessing pipeline to render them suitable for AI analysis. Images were first converted to 8-bit grayscale format and resized to a resolution of 128 × 128 pixels. To reduce illumination and contrast variability, Contrast Limited Adaptive Histogram Equalization (CLAHE) was applied with a clip limit value of 0.04.

### 2.6. Feature Extraction

To obtain discriminative image features, feature extraction was performed in two parallel channels. To represent local texture information, Gabor filters were applied to the images at orientations of 0°, 45°, 90°, and 135°, with a frequency parameter of 0.6. For each orientation, the mean and variance of the filter responses were calculated.

To describe the global shape and edge structure of the tooth, the Histogram of Oriented Gradients (HOG) method was employed using 16 × 16 pixel cells, a 2 × 2 block structure, and 9 orientation bins. All extracted features were combined into a single high-dimensional feature vector (Table 1).

### 2.7. Feature Normalization and Selection

The combined feature set was normalized using Z-score standardization (StandardScaler) to eliminate bias arising from different feature scales. To reduce overfitting and obtain a more discriminative representation, ANOVA F-value-based SelectKBest feature selection was applied, and the top 100 features (k = 100) yielding the best performance were retained for analysis.

### 2.8. Classification Model

The selected features were input into a Support Vector Machine (SVM) classifier with a Radial Basis Function (RBF) kernel. To account for potential class imbalance, balanced class weighting was employed. The model was designed as a decision-support tool rather than a replacement for clinical judgment, producing a binary classification output of “Normal” or “Indication present” for each panoramic radiograph.

### 2.9. Proposed AI-Based Computational Framework

The proposed AI-based evaluation algorithm aims to support extraction indication decisions for permanent first molars by analyzing panoramic radiographic image data within a structured computational pipeline. This approach is based on the combined evaluation of textural and geometric features that are critical to the clinical decision-making process.

In the initial stage, panoramic radiographs were converted to 8-bit grayscale format, resized to 128 × 128 pixels, and processed using CLAHE (clip limit = 0.04) to reduce illumination and contrast variability, thereby ensuring more consistent and comparable image analysis.

During the feature extraction stage, two parallel methods were applied to capture radiographic characteristics representing the developmental status of the permanent second molar and surrounding dentoalveolar structures. Gabor filters were used to characterize local texture features, while HOG descriptors were computed to reflect global shape and edge information.

The extracted Gabor and HOG features were merged into a single composite vector, normalized using Z-score standardization, and subjected to ANOVA F-value-based SelectKBest feature selection, yielding the most discriminative 100 features. These features were subsequently input into an RBF-kernel SVM classifier with class weighting, which generated a binary output of “Normal” or “Indication present” for each panoramic radiograph. The model was explicitly designed to support clinical decision-making rather than replace it.

### 2.10. Statistical Analysis

In this study, conventional parametric assumptions such as normal distribution were not applicable to the primary outcome, as the main objective was binary classification rather than hypothesis testing of continuous variables. Therefore, model performance was evaluated using distribution-independent machine learning metrics, including accuracy, precision, recall (sensitivity), F1-score, and the area under the Receiver Operating Characteristic curve (ROC–AUC). Classification outcomes were further examined using a confusion matrix.

In this classification context, effect size was interpreted in terms of discriminative performance measures (e.g., ROC–AUC) rather than traditional statistical effect size indices such as Cohen’s d. Age and sex distributions were compared between the Indication and Normal groups using standard comparative tests, and no statistically significant differences were observed between the groups (*p* > 0.05), indicating the absence of demographic confounding effects.

Given the limited sample size, this study was designed as an exploratory/pilot AI investigation. Therefore, no a priori power analysis was performed. A post hoc power analysis based on the observed overall classification accuracy (77.78%) demonstrated that the model performance was significantly above chance level (*p*_0_ = 0.50) in binary classification, with this superiority reaching statistical significance (*p* < 0.001), supporting the adequacy of the sample size for an exploratory classification task. All analyses were conducted using All analyses were conducted using Python (version 3.10.12, Python Software Foundation, Wilmington, DE, USA) with standard scientific computing libraries.

## 3. Results

The proposed hybrid Gabor–HOG–SVM model achieved an overall classification accuracy of 77.78%. Class-wise performance metrics are summarized in Table 2. For the Normal class, the precision, recall, and F1-score were 0.80, 0.74, and 0.77, respectively. For the Indication class, the corresponding values were 0.76, 0.81, and 0.79, indicating a balanced performance across both classes.

To assess the reliability of the reference classification, interobserver agreement analysis was performed using Cohen’s kappa coefficient, which yielded a value of 0.82, reflecting excellent agreement. This finding confirms the high consistency and reliability of the “Indication” and “Normal” labels used in the study.

The dataset comprised a total of 176 panoramic radiographs (88 Indication and 88 Normal cases). Model performance was evaluated on a randomly separated test set consisting of 27 images from each class (54 images in total).

**Figure 1:**
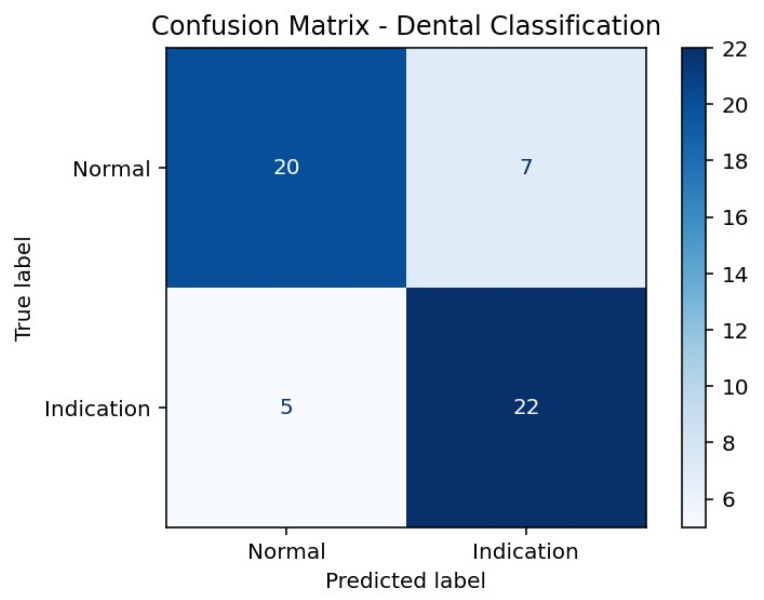
Confusion Matrix Heatmap of the Hybrid SVM Model.

According to the confusion matrix results presented in Table 3, the model correctly classified 20 of 27 Normal cases, while 7 Normal cases were incorrectly labeled as Indication. For the Indication class, 22 of 27 cases were correctly classified, whereas 5 cases were misclassified as Normal. This distribution indicates a higher sensitivity for detecting Indication cases, accompanied by a relatively higher rate of false-positive classifications in the Normal class (Figure 1).

Table 4 presents a comparative evaluation of different feature sets and classifier combinations. A Random Forest model using only HOG features achieved an accuracy of 60.61% with a macro F1-score of 0.60, while the HOG + LBP combination yielded a lower performance, with 48.48% accuracy and a macro F1-score of 0.48. The PCA + HOG-based SVM approach demonstrated moderate performance, achieving 55.56% accuracy and a macro F1-score of 0.56. All models were evaluated using the same training–test split. In comparison, the proposed hybrid Gabor + HOG + SVM approach outperformed all other configurations, achieving 77.78% accuracy and a macro F1-score of 0.78, thereby emerging as the most effective method.

**Figure 2:**
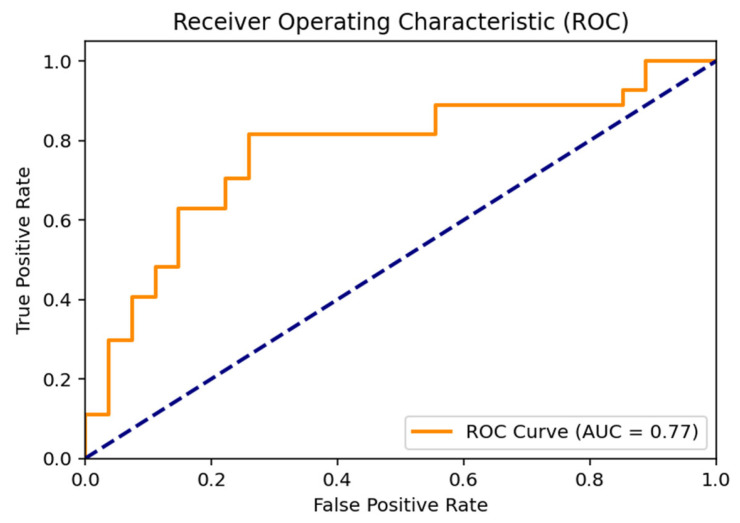
ROC Curve Analysis for Molar Tooth Classification. (The dashed diagonal line indicates random classification performance (AUC = 0.50)).

The Receiver Operating Characteristic (ROC) curve illustrating the discriminative performance of the proposed hybrid SVM model is positioned above the diagonal line representing random classification. The area under the curve (AUC) was calculated as 0.77, indicating a moderate-to-good discriminative ability between the Normal and Indication classes and supporting the overall robustness of the classification performance (Figure 2).

**Table 3:** Confusion matrix showing classification outcomes for extraction indication prediction.

Actual/Predicted	Predicted Normal	Predicted Indication
**Actual Normal**	20	7
**Actual Indication**	5	22

True Positive, correctly identified extraction-indicated cases; True Negative, correctly identified non-indicated cases.

**Table 4:** Comparative Analysis of Different Feature Sets.

Feature Extraction Method	Accuracy (%)	Macro Avg F1-Score
Random Forest (HOG only)	60.61%	0.60
Random Forest (HOG + LBP)	48.48%	0.48
SVM (PCA + HOG)	55.56%	0.56
**Proposed Hybrid (Gabor + HOG + SVM)**	**77.78%**	**0.78**

## 4. Discussion

Based on the findings of the present study, the null hypothesis was rejected. The AI-based evaluation model demonstrated a statistically meaningful ability to distinguish between extraction-indicated and non-indicated permanent first molars using panoramic radiographs.

In the present study, the extraction indication of permanent first molars in pediatric patients was evaluated using AI-assisted analysis of panoramic radiographs. Although numerous clinical and radiographic criteria related to the prognosis and extraction timing of permanent first molars have been described in the literature, these criteria are generally integrated through subjective assessments based on the clinician’s experience. In particular, biologically defined parameters such as Demirjian dental development stages are widely used in clinical decision-making; however, studies evaluating these decisions using objective, reproducible, AI-based classification models derived from panoramic radiographs remain limited. In this context, the present study proposes a novel decision-support approach aiming to predict “extraction indication present/absent” based on Demirjian E–F stages, thereby addressing an important gap in the literature.

Although Demirjian-based dental development stages provide a biologically grounded and widely accepted framework for estimating optimal extraction timing, it should be acknowledged that clinical extraction decisions are inherently multifactorial. Factors such as tooth restorability, occlusal relationships, arch length discrepancy, patient cooperation, and orthodontic treatment planning also play a critical role in real-world decision-making. Therefore, the present AI model should be interpreted as predicting a timing-based extraction indication window rather than providing a definitive clinical extraction recommendation. This distinction is essential to ensure appropriate clinical use and to avoid overinterpretation of model outputs.

Determining the extraction indication for permanent first molars is widely regarded as a complex clinical process requiring the simultaneous evaluation of multiple clinical and radiographic parameters. Due to their early eruption and susceptibility to caries and developmental defects, the prognosis of these teeth is often compromised; however, given their critical role as the key to occlusion, extraction decisions must be made with great caution. Arat Maden and Altun reported that, prior to extraction of permanent first molars with poor prognosis, factors such as restorability of the tooth, dental age, developmental stage of the permanent second molar, occlusal relationships, and existing orthodontic needs should be jointly considered. They further emphasized that the ideal extraction timing generally corresponds to the 8–10-year age range, during which spontaneous mesialization of the permanent second molar may be facilitated, leading to more favorable occlusal outcomes. Nevertheless, the largely experience-based and subjective nature of this multifactorial evaluation complicates the standardization of extraction indications. In this regard, AI-based decision support systems capable of simultaneously analyzing multiple radiographic features on panoramic images may help render this complex decision-making process more objective, reproducible, and consistent [15].

Panoramic radiographs allow comprehensive evaluation of dental and dentoalveolar structures in pediatric patients and serve as an important diagnostic tool for detecting pathologies and developmental anomalies that may not be evident during clinical examination. Bawazir et al. [16] reported that approximately 29.8% of pediatric patients exhibited one or more incidental dental anomalies on panoramic radiographs in their retrospective analysis. This finding highlights that panoramic imaging provides critical information not only for addressing current complaints but also for anticipating future occlusal disturbances and treatment needs. Early identification of anomalies affecting key teeth such as permanent first molars may contribute to more informed clinical decisions regarding extraction indications and timing—decisions that are often difficult to reverse [16].

However, because panoramic radiographs include multiple anatomical structures and are prone to superimposition, the evaluation process largely depends on the clinician’s experience [16]. In support of this limitation, Kaplan and Katı reported a high prevalence of positioning-related errors in digital panoramic radiographs, with 81.16% of images exhibiting at least one error, underscoring the susceptibility of panoramic imaging to operator- and patient-related variability. These findings further support the rationale for AI-assisted decision support systems aimed at reducing subjectivity and improving consistency in radiographic evaluation [17].

In recent years, the application of AI-based approaches in the interpretation of dental radiographs has emerged as a valuable tool to support clinicians in diagnosis and prognosis estimation. Lee et al. developed a deep learning-based convolutional neural network (CNN) using periapical radiographs and reported accuracy rates ranging from 73% to 83% in predicting extraction needs of periodontally compromised teeth. Their findings demonstrated that the performance of AI algorithms was comparable to that of experienced clinicians and was particularly accurate in detecting teeth with severe periodontal destruction. These results suggest that AI-assisted analysis of radiographic data can meaningfully contribute to clinical decision-making in evaluating tooth prognosis and extraction necessity [18].

During the mixed dentition period, multiple physiological mechanisms interact to establish ideal occlusion and esthetics. A thorough understanding of these processes enables early recognition and management of eruption-related problems, thereby helping to prevent prolonged, costly, and burdensome treatments later in life [19]. Consequently, timely extraction decisions for permanent first molars with poor prognosis are clinically critical for preserving arch symmetry and occlusal stability. It has also been suggested that, in selected cases, acceptable occlusal outcomes may be achieved through planned compensatory and/or balancing extractions, even in the absence of overt pathology.

Guidelines published by the Royal College of Surgeons of England indicate that space closure following extraction of maxillary permanent first molars may be relatively more favorable due to root morphology and mesial drift tendencies during eruption, whereas extraction timing in the mandible is more critical, with particular emphasis on the 8–10-year age range. Similarly, Kırzıoğlu and Ceyhan reported that, rather than relying solely on chronological age, greater emphasis should be placed on the root development stage of the permanent second molar when making extraction decisions for permanent first molars [20]. Given the multifactorial nature of this clinical decision, approaches aimed at standardizing panoramic radiograph-based decision-making processes may enhance consistency in clinical practice.

Previous guidelines have identified Demirjian stage E, corresponding to the onset of bifurcation calcification in the mandibular permanent second molar, as a marker for successful spontaneous space closure [21]. Osman Saraç also investigated the influence of Demirjian-based developmental stages of the permanent second molar on spontaneous space closure and reported significant differences between Demirjian stage E and more advanced stages (F–H), particularly in the mandible [22]. Conversely, Teo et al. emphasized that Demirjian stage E alone may not be sufficient to predict successful space closure and that additional factors such as second molar angulation and presence of the third molar germ may also play a determining role [23]. These findings highlight the limitations of relying on a single parameter in panoramic radiograph-based decision-making.

Accordingly, in the present study, Demirjian stages E–F were accepted as the appropriate time window for extraction, and labeling based on these stages constituted the reference standard for the AI model. This approach, which aligns with the ideal timing window reported in the literature, enabled the standardization of clinical decision-making based on biologically grounded criteria.

The widespread routine use and relative standardization of panoramic radiography provide an accessible and practical data source for decision support systems. Previous studies have suggested that teaching AI systems to recognize anatomy and pathology on dental images may constitute an important clinical decision support mechanism for clinicians [24,25,26,27]. In this context, the classification of panoramic radiographic features into “extraction indication present/absent” using AI in the present study should be viewed not as a replacement for clinical judgment, but rather as an objective and reproducible adjunct aimed at reducing observer-dependent variability and supporting the decision-making process.

The present findings should be interpreted in light of the dataset characteristics. Although the use of a relatively small and homogeneous dataset acquired from a single center and a single imaging device may enhance internal consistency, it also may introduce a potential risk of overfitting and limits the generalizability of the results. Model performance may vary when applied to panoramic radiographs obtained using different devices, acquisition protocols, or patient populations. Therefore, the reported results should be regarded as preliminary evidence supporting feasibility rather than definitive proof of clinical generalizability.

Beyond overall performance metrics, the clinical interpretation of classification errors warrants careful consideration, particularly in pediatric patients. False-positive classifications, in which a tooth without a true extraction indication is labeled as “Indication present,” may potentially lead to unnecessary early intervention if model outputs are misinterpreted or applied without adequate clinical validation. Conversely, false-negative classifications may result in delayed intervention, potentially reducing the likelihood of favorable spontaneous space closure. These considerations highlight the importance of interpreting AI-generated outputs within a comprehensive clinical context. Accordingly, the proposed model should be used strictly as a decision-support tool, with final extraction decisions remaining under the responsibility of the clinician.

With respect to model selection, a handcrafted feature-based machine learning approach using Gabor filters, HOG descriptors, and an SVM classifier was deliberately preferred over deep learning architectures. Although convolutional neural networks have demonstrated strong performance in large-scale dental imaging datasets, their effectiveness typically depends on extensive and diverse training data. In the context of a relatively limited and homogeneous dataset, deep learning models may be more susceptible to overfitting and reduced generalizability.

In contrast, classical machine learning methods combined with well-established handcrafted features allow more robust learning from smaller datasets, improved control over feature representation, and greater transparency in the decision-making process. Accordingly, the chosen SVM-based framework was considered more appropriate for this exploratory, proof-of-concept study and provides a solid methodological foundation for future investigations using larger multicenter datasets and deep learning approaches.

### Limitations and Future Directions

Several limitations of the present study should be acknowledged. First, this study was conducted using a retrospective design based on panoramic radiographs obtained from a single center. Although this approach ensured imaging consistency, it may limit the generalizability of the findings to data acquired using different devices, imaging protocols, or populations. In addition, external validation using multicenter datasets and heterogeneous imaging conditions is necessary to confirm the robustness and clinical applicability of the proposed model.

Second, extraction indication labeling was based solely on the Demirjian dental development stages of the permanent second molar. Other clinical and radiographic factors known to influence extraction decisions—such as second molar angulation, presence of the third molar germ, arch length discrepancy, and occlusal relationships—were not incorporated into the model. As a result, the proposed AI system may not fully capture the multifactorial nature of clinical decision-making.

Furthermore, the current model provides a binary classification outcome (“extraction indication present” or “absent”) and does not account for borderline or clinician-dependent decision scenarios. Therefore, it should be regarded strictly as a decision-support tool rather than a substitute for clinical expertise.

Future studies should focus on multicenter validation using larger and more diverse datasets, as well as the integration of additional clinical, radiographic, and orthodontic parameters. The incorporation of advanced deep learning architectures and longitudinal outcome data may further enhance model performance and clinical applicability.

## 5. Conclusions

This study demonstrates that AI-assisted analysis of panoramic radiographs can be used to support the evaluation of extraction indications for permanent first molars in pediatric patients. The proposed hybrid Gabor–HOG–SVM model achieved balanced and reproducible classification performance in distinguishing extraction-indicated and non-indicated cases based on Demirjian dental development stages.

From a clinical perspective, the findings suggest that panoramic radiographic features contain meaningful information relevant to extraction timing decisions, and that AI-based decision support systems may help reduce observer-dependent variability in complex pediatric dental assessments.

Importantly, the proposed model is not intended to replace clinical judgment, but rather to serve as an adjunctive tool that supports clinicians in making more objective and standardized evaluations. Further validation using multicenter datasets and the integration of additional clinical and radiographic parameters are required before such systems can be implemented in routine clinical practice.

## Figures and Tables

**Table 1:** Image preprocessing, feature extraction, feature selection, and classification parameters used in the proposed artificial intelligence framework.

Stage	Parameter	Value/Description
Preprocessing	Technique	CLAHE
	Clip Limit	0.04
	Resolution	128 × 128 Pixels (Grayscale)
Feature Extraction	Gabor Filter Orientations	0°, 45°, 90°, 135° (θ)
	Gabor Frequency	0.6
	HOG Orientations	9 Bins
	HOG Cells per Block	2 × 2
Feature Selection	Algorithm	SelectKBest (ANOVA F-value)
	Selected Features ($k$)	Top 100 features
Classification	Model	Support Vector Machine (SVM)
	Kernel Type	Radial Basis Function (RBF)

CLAHE: Contrast Limited Adaptive Histogram Equalization; HOG: Histogram of Oriented Gradients; SVM: Support Vector Machine; RBF: Radial Basis Function.

**Table 2:** Classification performance metrics of the proposed hybrid Gabor–HOG–SVM model.

Class	Precision	Recall	F1-Score	Support
Normal	0.80	0.74	0.77	27
Indicatio	0.76	0.81	0.79	27
Average/Total	0.78	0.78	0.78	54

Support indicates the number of test samples per class.

## Data Availability

The panoramic radiographs used in this study were obtained from the institutional archives of Batman University Faculty of Dentistry. Due to ethical and privacy restrictions, the data are not publicly available. The datasets may be made available from the corresponding author upon reasonable request.

## Abbreviations

The following abbreviations are used in this manuscript:

AI	Artificial Intelligence
AUC	Area Under the Curve
CLAHE	Contrast Limited Adaptive Histogram Equalization
CNN	Convolutional Neural Network
EF	Demirjian E–F developmental stages
F1-score	Harmonic mean of precision and recall
HOG	Histogram of Oriented Gradients
κ (Kappa)	Cohen’s kappa coefficient
LBP	Local Binary Patterns
PCA	Principal Component Analysis
RBF	Radial Basis Function
ROC	Receiver Operating Characteristic
SVM	Support Vector Machine
